# Standing on the Shoulders of the Giants: Dr. Kenneth Anderson

**DOI:** 10.46989/001c.92692

**Published:** 2024-02-05

**Authors:** Mohamad Mohty, Kenneth C. Anderson

**Affiliations:** 1 Sorbonne University, AP-HP, INSERM UMRs938, Paris, France; 2 Service d’Hématologie Clinique et de Thérapie Cellulaire, Hôpital Saint Antoine, AP-HP, Paris, France; 3 Department of Medical Oncology Dana-Farber Cancer Institute, Harvard Medical School, Boston, MA, USA

**Keywords:** Multiple myeloma, clinical hematology, pionneers

In a letter to Robert Hooke in 1675, Sir Isaac Newton made his most famous statement: “if I have seen further, it is by standing on the shoulders of giants.” This initiative of the International Academy for Clinical Hematology (IACH) aims to celebrate the achievements of leading experts and investigators whose work and research have helped to significantly advance the field of clinical hematology and establish the milestones and foundations of modern clinical hematology. This report represents a transcript of the interview given by Dr Kenneth C Anderson (KA) (**Figure 1**) on the 7th of November 2023, who responded to a series of questions asked by Dr Mohamad Mohty (MM).

**Figure 1.**
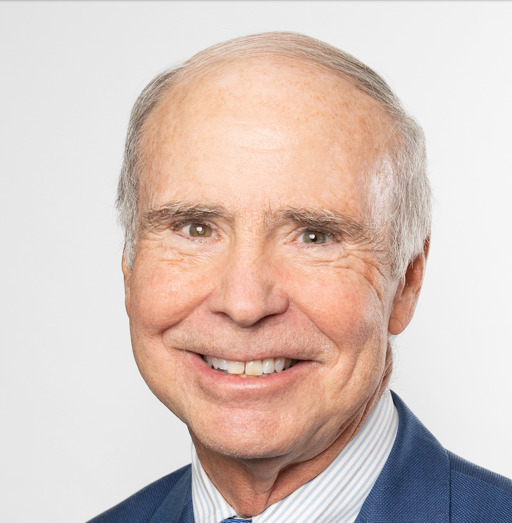
Dr Kenneth C Anderson

**MM**: Our honoree today is Dr Ken Anderson, Kraft Family Professor of Medicine, Harvard Medical School, and Director of the LeBow Institute for Myeloma Therapeutics and Jerome Lipper Multiple Myeloma Center at the Dana-Farber Cancer Institute in Boston, USA. He is a true giant in the field of multiple myeloma, a field which owes him a lot, which is why the Steering Committee of the IACH has unanimously elected him to be featured in this series. Dr Anderson, welcome, and thank you for joining us, it is really a great pleasure and an honor for us. How are you?

**KA**: I’m very well, and thank you so much for this honor.

**MM**: Usually in this broadcast, we start with a few personal questions and so I wonder whether you could share with us some information on your early childhood, early education, and how this may have played a role in you choosing to go into medicine and science.

**KA**: I grew up in a small town in Massachusetts in the USA and my mother was a nurse, which had a major impact in terms of my interest in science, but also focussed on trying to help people and make their world a better place. I was exposed to a general practitioner in our town, and intended to be a general practitioner or general care provider. I was the first person in our family to attend college or university where I majored in biology, while at the same time I did volunteer work as an orderly in the hospitals in Boston. During my upbringing and school years and then during university, I reaffirmed my interest in science and biology and my desire to help others, goals which seemed to be best achieved by a career in medicine. I do think to this day, Mohamad, that in medicine we are very privileged to have role models in life where we can see what kind of career would be rewarding for us-and luckily that’s what happened for me, early on.

**MM**: You mentioned this famous general practitioner who impressed you and you wanted to do like him. Were there any particular teachers at college/university that you thought were exemplary of the academic tradition and made you want to follow this path?

**KA**: There really were many individuals that I have been privileged to meet and learn from, both in terms of academic medicine, and honestly in how to live your life serving others. I would like to mention one in particular. As I said, I was destined to become a general practitioner in a small town, and then I went to medical school at Johns Hopkins in Baltimore, Maryland. My first teacher there was Dr Richard L. Humphrey, and the reason I mention him is that he was a myeloma researcher who worked on laboratory and animal models to try and develop new myeloma treatments. It really was eye opening for me because I had no interest nor experience in academic research. The two lessons that Dr Humphrey taught me I still carry with me today. One is to make science count for patients: do something in the laboratory or in the clinic that will change the diagnosis, prognosis, or treatment for the better for patients. The second most important lesson he taught me was to treat patients as family. I think these two tenets have motivated me every day, right until the present time, in terms of the patient interactions, we are so privileged to be able to do research that automatically helps patients; and then when you treat patients as family, you develop a long-term relationship and they do become as a second family. We satisfy our scientific and clinical curiosity, while at the same time we are forever changing things for the better for them and their families. I don’t think there is anything more rewarding than this.

**MM**: This is very beautiful and I do share the same philosophy and feel very humbled listening to what you are saying. Can I dig a little deeper into this myeloma story. I understand that at Johns Hopkins you discovered this myeloma research with Dr Humphrey but I’m guessing that at this time, myeloma was not the most ‘fashionable’ disease or the most developing in terms of innovation because I think this was the era where we were a little bit stuck, with treating almost everybody with melphalan and prednisone. So, what was the attraction of moving into this field and what was the status of the research into myeloma during this period?

**KA**: That’s a super question Mohamad, and I think it would be best to say that I saw this as a glaringly unmet need in medicine. The Hopkins experience really turned me on to academic research. I came up to the Dana-Faber Cancer Institute in Boston in 1980, and that was when monoclonal antibodies (mAbs) were brand new! No one here was working on myeloma, and I knew from my Hopkins years that research had advanced. There was melphalan and prednisone and combination chemotherapies such as VAD (vincristine, doxorubicin, dexamethasone), but patient survival was only 2-3 years. I first made a number of mAbs against plasma cells, recognized CD38 on myeloma cells in the early 80s, and studied the tumor microenvironment right from the start. This was a disease where it was relatively easy, as you know, to study not only the tumor cells, but also to try to figure out what in the microenvironment of the bone marrow nurtured myeloma, promoted its growth, and conferred drug resistance. Right from the start when we only had primitive models and understanding of biologic mechanisms, we were studying how to target the tumor cells with mAbs and how to understand the role of and target the microenvironment. You know this very well, Mohamad, because you have contributed immensely to the progress in multiple myeloma. In 1986, Dr Barlogie was doing high-dose therapy and stem cell transplantation in Little Rock, Arkansas, and we were doing it in Boston for the first time. We actually used mAbs back then to purge the bone marrow of patients receiving an autologous stem cell transplant. What then happened over time with this bench-to- bedside-to-bench research is that we exploited the scientific expertise that evolved over time. So, in the 90s cytokines, cell signalling, and genomics all came to the fore, and we tried in our myeloma model systems to understand the role of cytokines and the signalling pathways that were implicated in myeloma pathogenesis. We wanted to define the genomic abnormalities. In particular, we focussed on characterizing protein homeostasis in myeloma, and how to exploit it as a therapeutic target. Then, as you know, the rest is history. We and others since 2000 have helped develop multiple targeted therapies for our disease including proteasome inhibitors, the IMiDs (immunomodulatory drugs), CD38 mAbs etc. Since 2020 we have been part of the immune revolution in myeloma with CAR T-cells and bispecific T-cell engagers. When I came to DFCI in 1980, there was no one else working on myeloma, but now it has evolved to became a major focus: we have eight other professors at Harvard Medical School working in plasma cell diseases, and there are numerous myeloma research and clinical leaders all around the world who have come through our Center. All are now contributing to new scientific advances, and then making them count for patients.

**MM**: I didn’t realise that in the early 80s you were interested in mAbs and now I remember that CD38 was identified around 1978 and so it took more than 25 years to bring it into the clinic! You mentioned also, in parallel, what Dr Barlogie was doing with high-dose chemotherapy, and so can you tell us a little bit about the global interactions in the field of myeloma research in the 80s and 90s because you are also a believer in global collaboration, and I always mention and quote your saying “The United Nations Against Myeloma”. How was the situation in the 80s and 90s regarding interactions and collaborations, because today there are thousands of people interested in this field and doing a great job, but I guess it wasn’t the case back then?

**KA**: No, it wasn’t and unfortunately, myeloma patients really suffered with broken bones and repeated infections, as we didn’t have effective therapies. There wasn’t as much interest in the academic community since myeloma was thought of as a fatal and refractory disease. I think it’s fair to say that as the advances occurred, the awareness and interest in myeloma increased, with recognition that a collaborative team is needed to make bench-to-bedside advances. The team I am referring to is this collaborative model that you and I know well: patients are at the center, with people like you and I in academia doing either basic or clinical research and clinical trials; the biotech and pharma industry, who help with the novel agents and support the trials; the funders of cancer research including advocacy groups; and importantly, the regulators, who I do want to salute as being proactive on behalf of patients. Importantly, the patients are really the heroes and inspiration of this team. Why do I say this? Because, as the advances including the IMiDs and the proteasome inhibitors came, so the awareness and the interest increased, and the teams formed locally, nationally, and internationally. These teams are responsible for the fact that today we have 16 classes of new agents comprising 32 FDA approved treatments, and the outcome for myeloma has markedly improved. Each of us can make our individual contribution, but none of us alone can make a major difference without collaboration.

The other thing I’ll mention is the “United Nations Against Myeloma”. I did not anticipate this when I went into the field, but we have had the privilege of people coming through our Center who are now the leaders in myeloma not only in our country, but around the world. They remain personal friends to this day and also collaborators; consequently, the advances coming from our Center and others are being appreciated by patients all over the world. So, we are making science count for patients on the one hand, whilst having the legacy of the next generation of caregivers and researchers on the other. It is just a really wonderful reward, and I can’t think of anything better.

**MM**: You have built an amazing ‘war machine’ against myeloma which is about having the brains and resources. You mentioned the Jerome Lipper Multiple Myeloma Center at the Dana-Faber Institute and eight professors plus hundreds of colleagues who visited, trained, and now contribute. So, what is the secret or magic password, what did you do to attract all of these people to the field of myeloma and to give their best?

**KA**: Well, I don’t know if I have the secret, but this world is blessed with some truly motivated young investigators who are committed to making the world better for others. I’ve been amazed for over 40 years now to see these people come to Boston from all over the world, to work long hours for very little worldly rewards. They contributed to the initial preclinical and clinical trials of the IMiDs, proteasome inhibitors, many mAbs, HDAC (histone deacetylase) inhibitors, and immune therapies, and it continues to this day. I want to highlight that the caregivers here and around the world have had the reward of patients and families having a better outcome. When I started, Mohamad, I think you will remember that patients did very poorly. But what’s happened in the myeloma field and has been paralleled or modelled in other disease areas is truly remarkable-science has been translated to the bedsides, and today we can see the benefits to patients of the drugs we have helped to develop. We become long term friends with the patients and families because they are often living a normal lifespan. I think it’s the motivation of good people who see the needs and want to help others, and in this time of stress and strife in our world, it’s truly a beacon of hope.

**MM**: You mention your special relationship with patients and their families and that your mantra is about treating every patient as a family member and so my next question is how do your interactions with patients shape your research questions? You alluded to the process earlier, of the back and forth between bedside and laboratory.

**KA**: It’s a super question and I think it’s one of the unique opportunities of research and science. The truth is we have traditionally thought of medicine or research as going from the laboratory or animal models to the clinic. Finding an Achilles heel in the laboratory, then finding out how to exploit this in a treatment, and then doing clinical trials. Equally important is to learn from our patients, to listen to them in the clinic because they are the best teachers we’ll ever have. We also need to study the samples from our patients on treatment to understand how a medicine works or why it doesn’t work, and to suggest the next idea to overcome resistance to treatment and bring the next advance. Over and over again, we have had carried out such bench-to-bedside and back research. For example, the preclinical studies of the proteasome inhibitor bortezomib done in our Center and elsewhere in the 1990s showed the impact of inhibiting the proteasome in myeloma: direct toxicity; inhibiting the ability of the myeloma to survive and resist drugs in the microenvironment; and inhibiting the production of cytokines that promote tumor growth and survival. We went ahead from bench-to-bedside, and the first preclinical paper published by Teru Hideshima in 2001 about the proteasome inhibitor was just republished as a classic paper in oncology-I guess that means we’re getting old! In any event, that was an early bench-to-bedside translation to FDA approval in 2003. Twenty-three years later, we are still learning how the proteasome inhibitor works in the clinic. We now know that the proteasome inhibitor is actually also an immune-based treatment, as it triggers what is known as immunogenic cell death, an immune response in patients triggered by dying tumor cells which is beneficial in the clinic. This highlights how we must always be open to new understanding: we carry out clinical trials based on preclinical scientific rationale; however, the analysis of how it works in patients-the correlative science mechanisms in the tumor cell and in the microenvironment associated with response or not those are the lessons that allow us to go back and forth and keep making better next generation therapies. I’m so excited at the present time because we have always focussed on targeting the myeloma cell or in cancer more generally the tumor cell, but there is an awakening that we really need to think about the immune microenvironment and how to exploit it in our new treatments. It’s this iterative process of collaborative bench-to-bedside-to-bench back research that has fast forwarded progress in myeloma. .

**MM**: This is very inspiring. Please allow me to ask a question about your own family and about their contribution to your career, because with such dedication and energy, families are much involved, can you say a few words about this?

**KA**: We are truly blessed, and it is not an overstatement to say that we could not have done our research and clinical care without the devoted and loving support of our families. I mentioned that I was the first in our family to go to university, and my parents sacrificed incredibly for me to be able to do that. My wife Cynthia, who is an oncology nurse, and I have been married now for over 40 years and have three grown children. She has been the best pillar of support, the most understanding compass steering me in the right direction all these years, selflessly allowing me to do academic bench-to-bedside research. It is so important in life to have two ultimate goals: one is, what is your legacy in your own family? The second legacy is your academic family, which I have highlighted before. I can honestly say I’ve been so blessed with my wife Cynthia and our children Emily, Peter, and David (one’s a physician and two are in business) who all share the central core values of helping others make the world a better place. The medical influence in our own home has translated into careers for all of them which are people centered. Mohamad, you and I would not have been able to accomplish, either individually or as a myeloma academic community, any of our achievements if it wasn’t for our loving families.

**MM**: Thank you for this, and we are always very grateful to all of our family and friends for supporting our efforts. I have many more questions, but I will just ask two more. Is there anything you would have done differently, in hindsight? and what are your hopes for the future of hematology and what would you say to the younger generation?

**KA**: Honestly, I would not have done anything differently in hindsight. I don’t think I could ever have anticipated all that’s happened over the last four decades, but I also think that there’s no other career that could have coupled my interest in science with the reward of helping others more than the academic career that I have had. You have shared in multiple myeloma research-we have been very lucky, and we are very, very grateful.

In terms of the future of hematology, I don’t think it has ever been brighter: the advances in science, genomics, epigenomics, the understanding of the microenvironment, especially the immune potentials, are so extraordinary that diseases like myeloma are often a chronic illness; and I think cure is potentially on the horizon. I return Mohamad, to two quick points. One is the lesson I learned from Richard Humphrey in 1973 as a first-year medical student make science count for patients, do something to improve the diagnosis, prognosis, and treatment; and the second is treat patients as family. If you are a young aspiring researcher or caregiver, and use those two goals in your life, you will never go wrong.

I want to share with a treasured personal story to illustrate these goals. In 1986 I was doing autologous stem cell transplants in Boston, and the first patient who received one was an orthopaedic surgeon named Francesca Thompson. She did well for 13 years, which was a long time back then. My point about her story is that she wrote a book called ‘Going for the Cure’ published in 1989, which did a lot to increase the awareness of multiple myeloma and inspire researchers and patients all around the world. We became good friends and often lectured together: she would talk about the bone complications with myeloma, and I would talk about this new stem cell transplant treatment. We were together in front of an audience when someone asked her -you have written a book “Going for the Cure”, how will you know when you are cured? She replied “cure is growing old and dying from something else”. I think the message is that today many patients have myeloma as a chronic illness and die from something else. I do think some patients are cured in terms of the strict absence of disease, and the future is brighter still.

I would encourage the next generation: you can do wonderful science, better than ever before, and you can make it count for patients. Although we have lived through the golden years of myeloma, the next years are going to be the curative years for this and many other diseases. So, making the world a better place and treating patients as family-using these as your goals, you’ll never go wrong.

**MM**: Thank you so much Ken, this was a really beautiful and very touching summary. I learnt a lot from this interview, and on behalf of the myeloma community I would like to express our deepest gratitude for everything you have done to advance the field and everything you continue to do; as you said, it’s about curing more and more patients and we are very close to this goal, so thank you so much to the giant Ken Anderson, and I hope to catch up with you soon.

**KA**: Thanks so much.

## ETHICAL APPROVAL

Not applicable.

## CONSENT TO PARTICIPATE/INFORMED CONSENT

Not applicable.

## CONSENT FOR PUBLICATION

Not applicable.

## COMPETING INTERESTS/CONFLICT OF INTEREST

Not applicable.

## AUTHORS’ CONTRIBUTION – CREDIT TAXONOMY

Conceptualization: Mohamad Mohty (Equal), Kenneth C. Anderson (Equal). Writing – original draft: Mohamad Mohty (Lead). Writing – review & editing: Mohamad Mohty (Equal), Kenneth C. Anderson (Equal).

